# Trends in the Prevalence of Hepatitis B Virus, Hepatitis C Virus, and HIV Infections in Iranian Patients with Hereditary Bleeding Disorders

**DOI:** 10.3390/pathogens12040555

**Published:** 2023-04-04

**Authors:** Azar Gharoonpour, Saeideh Maleki, Hamid Sharifi, Seyedehsara Osia, Heidar Sharafi, Maryam Keshvari

**Affiliations:** 1 Cancer Biology Research Center, Tehran University of Medical Sciences, Tehran 1419733141, Iran; 2 Middle East Liver Diseases (MELD) Center, Tehran 1598976513, Iran; 3 HIV/STI Surveillance Research Center, WHO Collaborating Center for HIV Surveillance, Institute for Futures Studies in Health, Kerman University of Medical Sciences, Kerman 7619833477, Iran; 4 School of Nutrition, Toronto Metropolitan University, Toronto, ON M5B 2K3, Canada; 5 Blood Transfusion Research Centre, High Institute for Research and Education in Transfusion Medicine, Tehran P.O. Box 1449613111, Iran

**Keywords:** blood safety, hemophilia A, hepatitis B, hepatitis C, HIV, Iran

## Abstract

Background: Patients with hereditary bleeding disorders (HBDs) have always been vulnerable to transfusion-transmitted infections (TTIs) such as hepatitis B virus (HBV), hepatitis C virus (HCV), and human immunodeficiency virus (HIV) infections due to being regular recipients of blood and blood products. This study aimed to detect the trends in the prevalence of HBV, HCV, and HIV infections by birthyear in Iranian patients with HBDs to show the efficacy of national interventions implemented to administrate control and to prevent these infections, i.e., blood safety, newborn HBV vaccination, and safe replacement treatments. Methods: In this retrospective study, the trends in the prevalence of hepatitis B core antibody (HBcAb), HCV antibody (HCV-Ab), and HIV antibody (HIV-Ab) in Iranian patients with HBDs born before 2012 were assessed using patients’ clinical archives. The determinants of HBV, HCV, and HIV infections were investigated in bivariable and multivariable logistic regression analyses. Results: Out of 1475 patients with HBDs, most were male (87.7%) and diagnosed with hemophilia A (52.1%) and severe bleeding disorder (63.7%). The prevalence of HBcAb, HCV-Ab, and confirmed HIV-Ab was 22.9%, 59.8%, and 1.2%, respectively. The trends in HBcAb, HCV-Ab, and HIV-Ab were all decreasing by birthyear and reached a stable level of 0% for patients with birthyears in 1999, 2000, and 1984, respectively. In multivariable analysis, birthyear was significantly associated with HBcAb prevalence. In the multivariable analysis, type of HBD; birthyear; bleeding severity; histories of receiving packed cells, fresh frozen plasma, and cryoprecipitate before 1996; and history of receiving factor concentrate before 1997 were highly associated with the prevalence of HCV-Ab. Moreover, in the bivariable analysis, birthyear and type of HBD were associated with HIV-Ab prevalence. Conclusion: This study demonstrated the decreasing trends in HBV, HCV, and HIV seroprevalence in Iranian patients with HBDs following preventive interventions such as HBV vaccination, blood safety measures, and the provision of safe replacement treatments.

## 1. Introduction

Transfusion-transmitted infections (TTIs) contribute significantly to the morbidity and mortality of patients with hereditary bleeding disorders (HBDs) [1]. Hereditary bleeding disorders occur due to the absence or deficiency of one or more clotting factors. The three most common HBDs are hemophilia A (factor VIII deficiency), hemophilia B (factor IX deficiency), and von Willebrand disease (VWD). The bleeding episodes of these patients are treated using fresh frozen plasma (FFP), cryoprecipitate, and plasma-derived or recombinant factor concentrates. Although using these products led to a significant reduction in the morbidity and mortality following bleeding episodes in patients with HBDs, these replacement therapies have been associated with the transmission of TTIs, especially viral infections such as hepatitis B virus (HBV), hepatitis C virus (HCV), human immunodeficiency virus (HIV), and human T-lymphotropic virus (HTLV) infections [2,3]. The transmission risk of TTIs increases with plasma-derived factor concentrates made from large pools of donors. Since HCV- and HIV-infected patients carry a high level of plasma viral load, most of the concentrated factors derived from them are infected with HCV and/or some with HIV, too [4]. Most patients with HBDs were treated with locally provided FFPs and cryoprecipitates in Iran between 1970 and 1986, the period with the highest prevalence of HCV and HIV infections among these individuals. However, a small portion of these patients was receiving untreated factor concentrates [5,6]. The Centers for Disease Control and Prevention (CDC) estimates that 60–90% of people with hemophilia who used clotting factors before 1987 were heavily exposed to HCV infection through contaminated blood products [7]. In a systematic review, the seroprevalence of HCV among patients with HBDs in the eastern Mediterranean countries by pooled estimation was 48.27% (95% CI: 36.12–60.43). In Iran, the seroprevalence of HCV among patients with hemophilia varied from 13.3% to 80.5%, and the pooled estimation was 48.07% (95% CI: 35.66–60.48) [2]. Many individuals with HBDs were infected with HIV through treatment with contaminated blood and blood components before there was an HIV screening test for these products. According to the World Federation of Hemophilia report on the annual global survey in 2020 and 2021, the total population of individuals living with HBDs and HIV in Iran was 26 (twenty-four hemophiliacs, one VWD, and one in other types of bleeding disorders) [8]. Hepatitis B virus infection is less prevalent than HCV infection among patients with HBDs. This is because the donor screening for HBV was started earlier, and HBV vaccination played a significant role in this difference. A study on a cohort of patients with HBDs in the US demonstrated that 30% of them were seropositive for HBV markers [9].

The journey of blood safety in Iran started with donor selection and screenings of donors for hepatitis B surface antigen (HBsAg) in 1974, and screenings for HIV antibody (HIV-Ab) and HCV antibody (HCV-Ab) were implemented in 1989 and 1996, respectively [10]. Vaccination against HBV was launched for patients with HBDs in the 1990s and the national newborn HBV vaccination was established in 1993 [11]. The investigations upon solvent/detergent (SD) that started in 1982 brought results for solvent/detergent-treated factor VIII in 1985 in the USA, and the license to perform was granted to the main companies [12].

Numerous studies have been carried out about the prevalence of TTIs among Iranian patients with HBDs, but none of them addressed the trends of TTIs in Iranian patients with HBDs and the effectiveness of national interventions for the prevention of TTIs in Iranian HBDs [13,14,15]. The main objective of this study is to evaluate the trends in the seroprevalence of HBV, HCV, and HIV by birthyear in Iranian patients with HBDs to show the effectiveness of interventions for the prevention of HBV, HCV, and HIV transmission, i.e., blood safety, newborn HBV vaccination, and safe replacement treatments, among Iranian patients with HBDs. As the secondary objective, the overall prevalence of HBV, HCV, and HIV biomarkers such as hepatitis B core antibody (HBcAb), HBsAg, HCV-Ab, and HIV-Ab in Iranian patients with HBDs was assessed, and the magnitude of the risk caused by receiving unsafe blood and blood products was measured. Finally, this study highlights the role of diagnostic, prophylactic, and treatment action plans in line with the elimination of HBV and HCV infections in Iran.

## 2. Methods

### 2.1. Study Design and Population

The main data of this retrospective study were collected in the summer and fall of 2015 from available clinical records of patients with HBDs who were referred to the Iranian Comprehensive Hemophilia Care Center (ICHCC). The Iranian Comprehensive Hemophilia Care Center is a national referral healthcare center for Iranian patients with HBDs supervised by the Iranian Hemophilia Society (IHS), providing services based on international standards. The inclusion criteria were patients who were (a) diagnosed with HBDs such as hemophilia A, hemophilia B, VWD, etc.; (b) born before 2012; and (c) with available results of testing for HCV-Ab and HIV-Ab after 2005. The protocol of the study and the use of data collected in 2015 was confirmed by the ethics committee of Kerman University of Medical Sciences with approval code IR.KMU.REC.1401.394 in December 2022. Since this retrospective study was designed based on the collection of data from clinical archives, obtaining informed consent was waived. The authors assert that all procedures contributing to this study complied with the ethical guidelines of the 1975 Declaration of Helsinki.

### 2.2. Data Collection

The following data were collected from the patients’ records: birthyear; sex; type of bleeding disorder (hemophilia A, hemophilia B, VWD, and other disorders); the severity of the bleeding disorder (mild, moderate, and severe); histories of receiving packed cells, FFP, cryoprecipitate, and factor concentrates; and the latest results of HBcAb, HBsAg, HCV-Ab, and HIV-Ab tested after 2005. In cases with reactive HIV-Ab in screening, the results of HIV-Ab confirmation using a western blot test were collected from the patients’ records. In cases with reactive HCV-Ab, the available results for HCV RNA by RT-PCR and HCV genotyping before treatment were gathered. All the above-mentioned biomarkers for HBV, HCV, and HIV were tested in accredited diagnostic laboratories as a part of the screening and clinical management of these infections in patients referred to the ICHCC.

### 2.3. Measures

#### 2.3.1. Hepatitis B Virus Measures

The results of HBcAb were considered as an individual’s history of hepatitis B infection. Since the two effective interventions for the prevention of HBV infection in patients with HBDs in Iran were the implementation of HBV screening in blood donors in 1974 and newborn HBV vaccination in 1993, these two years were selected as cut-offs for stratification by birthyear of the study participants in the evaluation of parameters influencing exposure to HBV. Given that the screening of blood donors for HBV was implemented in 1974, receiving packed cells, FFP, or cryoprecipitate before 1974 was included as a risk factor for HBV infection. For factor concentrate, 1993 was selected for dichotomization since HBV vaccination brings a high level of protection against HBV infection.

#### 2.3.2. Hepatitis C Virus Measures

The results of HCV-Ab were used for the history of HCV infection. Since two pivotal mediations for HCV protection were the screening of HCV-Ab in blood donors in 1996 and safe replacement therapy enforcement in 1997, for the stratification of patients’ birthyears, the 1997 cut-off (including 1996 impacts) was considered as an ideally suited date for assessing the parameters influencing HCV seroprevalence. Given that the screening of donors for HCV-Ab was implemented in 1996, receiving packed cells, FFP, or cryoprecipitate before 1996 was counted as a risk factor for HCV infection. For the history of receiving factor concentrate, 1997 was preferred for dichotomization since the implementation of treatment using safe replacements conferred an increased level of protection against HCV infection.

#### 2.3.3. Human Immunodeficiency Virus Measures

The results of HIV-Ab confirmed by western blot were used as the history of HIV infection. As the screening of blood donors for HIV in 1989 and the implementation of safe replacement therapy in 1997 were of value in HIV infection control, they were selected for birthyear stratification. For receiving packed cells, FFP, and cryoprecipitate, the 1989 cut-off (date for implementation of HIV screening in blood donors) was considered. For the history of receiving factor concentrate, 1997 was considered for dichotomization because the performance of safe replacement therapy granted remarkable protection against HIV infection.

### 2.4. Data Analysis

Categorical variables are presented as frequencies and percentages. Continuous variables are expressed as medians and interquartile ranges (IQR). For the calculation of the 95% confidence interval (CI) of proportion, one-sample binomial success rate (Clopper–Pearson) was used. The trends in the prevalence of HBV, HCV, and HIV infections by birthyear were analyzed using a generalized linear model for binary outcomes. The determinants of infection were analyzed using a bivariable binary logistic regression or Fisher’s exact test. The effects of covariates with *p* < 0.1 in the bivariable analysis were adjusted using multivariable binary logistic regression. *p* < 0.05 was considered statistically significant. All statistical analyses were carried out using SPSS version 22 (IBM SPSS) and the statistical graph was generated using GraphPad Prism 6.

## 3. Results

### 3.1. Characteristics of the Study Population

In this retrospective study, a total of 1475 patients with HBDs were included, among whom 87.7% were male. Most patients suffered from hemophilia A (52.1%) and severe HBD (63.7%). Among the study population, 90.6% had a history of receiving factor concentrates, 62.6% had a history of receiving cryoprecipitate, 61.9% had a history of receiving fresh frozen plasma, and 47.5% had a history of receiving packed cells. The patients’ characteristics are summarized in Table 1.

### 3.2. Hepatitis B Virus Infection in Patients with Hereditary Bleeding Disorders

**Figure 1:**
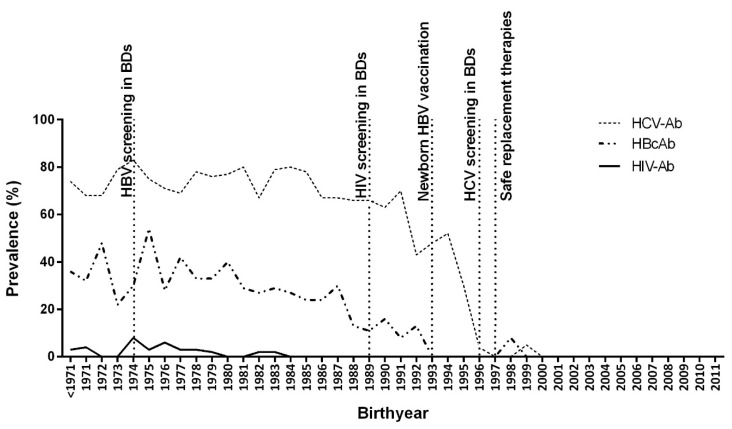
Trends in prevalence of HBcAb, HCV-Ab, and confirmed HIV-Ab by birthyear of patients with hereditary bleeding disorders. Generalized linear model for binary outcomes (adjusted for sex and type and severity of bleeding disorder): HBcAb, β: −0.054, 95% CI: −0.065–−0.042, *p* < 0.001; HCV-Ab, β: −0.116, 95% CI: −0.132–−0.100, *p* < 0.001; confirmed HIV-Ab, β: −0.070, 95% CI: −0.104–−0.036, *p* < 0.001.

The prevalence of HBcAb among patients with HBDs was 22.9% (95% CI: 20.7–25.3). The prevalence of HBcAb was 25.3% in patients with hemophilia A, 21.6% with hemophilia B, 20.9% with VWD, and 11.8% with other bleeding disorders. A gradual decline in the trend of HBcAb prevalence by birthyear in those born after 1974 showed that HBV prevalence was influenced by the implementation of HBV screenings in blood donors, and after launching the newborn HBV vaccination in 1993, the prevalence of HBcAb by birthyear dropped, indicating that the newborn HBV vaccination had a crucial impact on HBV prevalence (Figure 1).

In the bivariable analysis of determinants of HBcAb prevalence, birthyears from 1974–1993 and birthyears during and after 1993 (vs. before 1974, OR: 0.62 and 0.01) were associated with a lower prevalence of HBcAb. Moreover, histories of receiving packed cells before 1974 (OR: 2.27), FFP before 1974 (OR: 2.32), cryoprecipitate before 1974 (OR: 2.72), and factor concentrate before 1993 (OR: 8.17) were associated with a higher prevalence of HBcAb. Hemophilia A (vs. other disorders/VWD, OR: 1.44) was also associated with a higher prevalence of HBcAb in the study population (Table 2). In multivariable analysis, only birthyears during and after 1993 (aOR: 0.04) remained a statistically significant determinant of HBcAb positivity in patients with HBDs (Table 2).

In the study population, the HBsAg prevalence was 1.3% (95% CI: 0.8–2.0), and among 309 individuals with HBcAb, 19 (6.1%) were found to be positive for HBsAg. The prevalence of HBsAg was 1.6% in hemophilia A, 1.2% in hemophilia B, 0.7% in VWD, and 1.3% in other bleeding disorders.

### 3.3. Hepatitis C Virus in Patients with Hereditary Bleeding Disorders

Hepatitis C virus antibody was detected in 59.8% (95% CI: 57.2–62.3) of patients with HBDs. The prevalence of HCV-Ab was higher in patients with hemophilia A (70.8%) and hemophilia B (66.3%) than in those with VWD (34.1%) and other bleeding disorders (24.1%). The trend of HCV-Ab prevalence by birthyear in patients born before 1989 experienced a fluctuation of around over 70%. However, after the implementation of HIV screenings of blood donors in 1989, the HCV-Ab trend showed a decrease and finally, after the implementation of HCV screenings of blood donors, reached 0% (Figure 1).

The bivariable analysis presented many parameters associated with HCV seroprevalence, including male sex (OR: 3.76), birthyears during and after 1997 (OR < 0.01), hemophilia A and hemophilia B (vs. VWD/other HBDs, OR: 5.16 and 4.17), moderate and severe HBDs (vs. mild, OR: 2.41 and 4.28), the history of receiving packed cells before 1996 (OR: 3.78), the history of receiving FFP before 1996 (OR: 7.19), the history of receiving cryoprecipitate before 1996 (OR: 8.85), and the history of receiving factor concentrates before 1997 (OR: 29.14) (Table 3). In multivariable analysis, birthyears during and after 1997 (aOR: 0.01), hemophilia B (vs. VWD/other HBDs, aOR: 2.91), moderate and severe HBDs (vs. mild, aOR: 2.20 and 5.99), the history of receiving packed cells before 1996 (aOR: 1.50), the history of receiving FFP before 1996 (aOR: 2.21), the history of receiving cryoprecipitate before 1996 (aOR: 3.96), and the history of receiving factor concentrates before 1997 (aOR: 3.01) remained as statistically significant determinants of HCV-Ab prevalence in HBDs (Table 3).

Among 882 individuals with HCV-Ab, 859 (97.4%) had available results for HCV-RNA assessment in their medical records, consisting of 793 (92.3%) with detectable HCV-RNA (current HCV infection) and 66 (7.7%) without HCV-RNA (HCV spontaneous clearance). Among the 793 with positive results for HCV-RNA, 572 (72.1%) had results of HCV genotyping, consisting of three hundred and sixty-five (63.8%) HCV-1, nine (1.6%) HCV-2, one hundred and eighty-five (32.3%) HCV-3, three (0.5%) HCV-4, nine (1.6%) mixed HCV genotypes, and one (0.2%) untypable HCV genotype.

### 3.4. Human Immunodeficiency Virus in Patients with Hereditary Bleeding Disorders

Using ELISA for the evaluation of HIV-Ab, 23 (1.6%) were found to be reactive to HIV-Ab. Among these 23 patients with reactive HIV-Ab, testing of HIV-Ab using western blot resulted in 18 (78.3%) confirmations of HIV infection. Among the study population, 1.2% (95% CI: 0.7–1.9%) were found with confirmed HIV-Ab. The prevalence of confirmed HIV-Ab was 1.0% in hemophilia A, 3.1% in hemophilia B, and 0% in VWD and other bleeding disorders. According to the confirmed HIV-Ab trend by birthyear among patients with HBDs, the highest prevalence of HIV-Ab was observed in those born in 1974, followed by a decreasing trend, reaching no new individual with HIV-Ab in those born after 1983 (Figure 1).

In the analysis of determinants of HIV-Ab prevalence, birthyears during and after 1989 (OR: 0.12), and hemophilia B (vs. VWD/other HBDs, OR: 12.06) were associated with HIV-Ab prevalence in patients with HBDs (Table 4).

## 4. Discussion

Despite the worldwide availability of the synthesized concentrates of coagulation factors, there have been limitations in the provision of effective bleeding management among patients with HBDs in developing countries. The literature has demonstrated HCV as the most prevalent TTI among subjects with hemophilia. The seroprevalence of HCV among the general population of Iran was estimated at 0.3% [16]. The results of the current study showed that 60% of the patients were HCV-seropositive. In a cross-sectional study among hemophilic patients registered in the Iran Hemophilia Society Center in Tehran carried out by Mousavian et al. [17] from 2003 to 2005, the prevalence of HCV-Ab was reported as 72.3%. Furthermore, a comprehensive review evaluating the epidemiology of HCV in Iran conducted by Mahmud et al. [16] indicated that HCV prevalence among hemophiliacs ranged from 6.0% to 90.0%, with a median of 54.0%. The results of the current study showed that HCV-Ab prevalence was steady until 1991 and sharply declined after 1991. This decline may be because of multiple interventions since 1989, including the implementation of HIV-Ab/Ag screenings in blood donors in 1989 that led to the removal of HIV-HCV co-infected donors in addition to improvements in the donor deferral system in line with universal standards. Moreover, the developments that occurred in donor selection strategies such as screening by abnormal ranges of liver enzymes and venereal disease research laboratory (VDRL) tests resulted in the removal of donors with the probability of HCV infection, leading to a steady reduction in the transmission of HCV before the implementation of HCV screenings in blood donors in 1996 [18,19]. This finding is consistent with the previous studies aiming to determine the interventions effective in reducing HCV prevalence [4,9,20].

In the present study, the patients with moderate or severe clotting factor deficiencies had a higher prevalence of HCV infection than those diagnosed with mild bleeding disorders. In a study by Goedert et al. [20], there was a high HCV infection incidence in moderate and severe hemophilic cases with 11.7%/year in 1970 and 17.2%/year in 1968. In another study carried out by Carmo et al. [21], the HCV-Ab positivity in Brazilian male patients with hemophilia from 1985 to 2015 was assessed and showed that 12.6% of patients with mild hemophilia were seropositive for HCV-Ab while 39.6% of those with moderate or severe hemophilia were reactive for HCV-Ab. The prevalence of HCV-Ab was significantly higher in the current study population who received blood and blood products (FFP, cryoprecipitate, and factor concentrate) before 1996/1997 compared to the patients who received them after 1997. A study in Zahedan, a city in the southeast of Iran, illustrated that 35% of the patients with hemophilia who were treated with plasma products before 1996 were HCV-seropositive, while HCV-Ab was detected in 6% of those treated with plasma products after 1996 [22]. Additionally, a similar study conducted in Taiwan demonstrated that 80% of the patients with hemophilia born before 1987 were infected with HCV; however, the prevalence among transfusion-dependent individuals significantly decreased after the implementation of preventative measures [23]. Although all blood and blood products had a significant role in HCV transmission in the current study, cryoprecipitate and clotting factor concentrates had the most detrimental effects. A cohort study by Geodert et al. [20] indicated an increase in HCV incidence upon receiving FFP and cryoprecipitate, especially before clotting factor concentrates were licensed in the 1970s among patients with HBDs.

In the current study, 7.7% of HCV-seropositive patients spontaneously cleared HCV. According to Yee et al. [24], 9% of the total hemophilic population of their study cleared HCV spontaneously. In the above-mentioned retrospective study, a large percentage of the patients in the spontaneous HCV clearance group had non-severe hemophilia and were exposed to less amounts of concentrated factors compared to their counterparts. Furthermore, Zhang et al. [25] indicated spontaneous HCV clearance in 27% of subjects with hemophilia. The low percentage of spontaneous HCV clearance in our study compared to similar studies can be justified by the study recruitment having been in a specialized clinic for antiviral therapy of HCV infection, resulting in a bias toward more patients with current HCV infection.

In this study, the prevalence of HBcAb, the marker of HBV infection, was 22.9%. A similar study reported that the prevalence of HBcAb among patients with HBDs in Iran was 44% [26]. The noticeable difference in the prevalence of HBcAb between the two studies may be due to the temporal differences. In the current study, the percentage of individuals born after 1993, the year newborn HBV vaccination started in Iran, was higher than in the study by Nassirtoosi et al. [26]; hence, a higher number of individuals in our study were immune to HBV. As our study indicated that the prevalence of HBcAb steadily decreased upon the introduction of HBV screenings in blood donors in 1974, the implementation of HIV-Ab screenings in blood donors in 1989 further aided the reduction of the prevalence of HBcAb by removing high-risk individuals from the donor pool. Finally, the national implementation of newborn HBV vaccination in 1993 markedly reduced the prevalence of HBcAb. According to a meta-analysis, the prevalence of the current HBV infection (HBsAg seropositivity) in Iran decreased from 3.02% in 2000 to 1.09% in 2016 in the general population [27]. In another meta-analysis, the prevalence of HBcAb in the Iranian general population was estimated as 13.59% (95% CI: 12.92–14.29), and the prevalence of HBsAg was considered to be 1.79% (95% CI: 1.67–2.32) [28]. Comparing the prevalence of HBcAb and HBsAg in the general and hemophilic populations, after the implementation of HBV vaccination among these individuals, the prevalence of this infection in both populations is similar. This finding demonstrates the effectiveness of these safety measures in reducing HBV infection among the hemophilic population.

In the current study, 1.2% of the patients were HIV-seropositive. The latest United Nations Programme on HIV/AIDS (UNAIDS) report for Iran revealed that the count of Iran’s HIV population in 2019 stood at 59,314 (Range: 32,685–125,636) [29]. A review article reported a 0–2.3% prevalence of HIV infection among patients with hemophilia in different provinces of Iran. This prevalence of HIV seropositivity documented in Iranian multi-transfused patients is noticeably less than in the same population of patients in western countries with 39–90% HIV prevalence [5]. In the current study, all HIV-seropositive patients had a history of receiving factor concentrate before 1997 while none of them had a history of receiving blood and other blood products before 1989. Based on the above-mentioned findings, we postulate the factor concentrates before 1997 as the source of HIV infection in Iranian patients with HBDs.

This study demonstrated the remarkable effectiveness of the national interventions for the prevention of TTIs in Iranian patients with HBDs; however, we did not address the interventions and strategies for complementary diagnosis, antiviral therapy, and management of these infections. In Iran, patients with HBDs have been recognized as a target group for the diagnosis and management of HCV infection for more than 20 years. They have been regularly screened for HCV infection and directed to treatment; however, the first antiviral therapies used for the treatment of HCV in HBDs in Iran were interferon (IFN)-based treatments accompanied by no more than a 50% chance of curing the patient [30]. Hopefully, the anti-HCV treatments introduced recently are IFN-free with more than a 95% chance of achieving a sustained virologic response in patients with HBDs [31]. With the availability of new diagnostic and therapeutic modalities, the World Health Organization (WHO) has urged to plan for the elimination of HCV and HBV and 90–90–90 targets for the control of HIV by 2030 [32,33].

The strengths of this study include its large sample size and the method of data extraction and analysis which allowed the portrayal of the incidence and trend of TTIs among Iranian patients with hemophilia for the first time. The limitations of this study include its retrospective design and the possible inaccuracies associated with relying on patients’ memories to recall events. The current study is mainly limited by using the birthyear for trends of TTIs since the exact time the patients contracted the viral infections is unknown. Moreover, the study setting where the data were gathered was a referral center, leading to the possibility of selection bias in this study.

In conclusion, the safety measures implemented, including HBV, HIV, and HCV screening in blood donors, newborn HBV vaccination, and safe replacement therapies, have effectively decreased the prevalence of TTIs among patients with HBDs. Moreover, the employment of HCV antiviral therapies has allowed the micro-elimination of hepatitis C among these individuals. Despite being the safest intervention in treating of hemophilia, recombinant hemophilic factors are not always available in developing countries. Thus, the government should invest in improving access to this intervention or at least maintain the proper surveillance of the blood donation system and standard screening methods.

## Figures and Tables

**Table 1:** Characteristics of the study population.

**Variables**		**Values**
**Sex, n (%)**		
	Male	1293 (87.7)
	Female	182 (12.3)
**Birthyear**		
	Median	1983
	Rang (min–max)	1925–2011
**Type of hereditary bleeding disorder, n (%)**		
	Hemophilia A	768 (52.1)
	Hemophilia B	326 (22.1)
	Von Willebrand disease	302 (20.5)
	Other disorders	79 (5.3)
**Bleeding severity, n (%)**		
	Mild	264 (22.7)
	Moderate	158 (13.6)
	Severe	739 (63.7)
History of receiving packed cells, n (%) *		696 (47.5)
History of receiving fresh frozen plasma, n (%) *		907 (61.9)
History of receiving cryoprecipitate, n (%) *		916 (62.6)
History of receiving factor concentrates, n (%) *		1333 (90.6)

Abbreviations: n, number; y, year; IQR, interquartile range. * Less than 10% missing data.

**Table 2:** Bivariable and multivariable logistic regression analysis of the determinants of HBcAb prevalence in patients with hereditary bleeding disorders.

	Individuals with HBcAb, n (%)	Individuals without HBcAb, n (%)	OR (95% CI)	*p*-Value *	Adjusted OR (95% CI)	Adjusted *p*-Value **
**Sex, n (%)**						
Female	35 (11.3)	126 (12.1)	Ref			
Male	274 (88.7)	913 (87.9)	1.80 (0.73–1.61)	0.703		
**Birthyear, n (%)**						
Before 1974	116 (37.5)	208 (20.0)	Ref		Ref	
1974–1993	191 (61.8)	555 (53.4)	**0.62 (0.47–0.82)**	**<0.001**	1.07 (0.54–2.15)	0.842
During and after 1993	2 (0.7)	276 (26.6)	**0.01 (<0.01–0.05)**	**<0.001**	**0.04 (<0.01–0.19)**	**<0.001**
**Type of hereditary bleeding disorder, n (%)**						
Other disorders/VWD	66 (21.4)	280 (27.0)	Ref		Ref	
Hemophilia A	180 (58.2)	531 (51.1)	**1.44 (1.05–1.98)**	**0.025**	1.22 (0.86–1.73)	0.274
Hemophilia B	63 (20.4)	228 (21.9)	1.17 (0.80–1.73)	0.421	1.06 (0.69–1.61)	0.800
**Bleeding severity, n (%)**						
Mild	50 (20.3)	195 (23.9)	Ref			
Moderate	27 (11.0)	116 (14.2)	0.91 (0.54–1.53)	0.716		
Severe	169 (68.7)	504 (61.9)	1.31 (0.92–1.87)	0.140		
**History of receiving packed cells before 1974, n (%)**						
No	227 (73.7)	907 (88.1)	Ref		Ref	
Yes	81 (26.3)	123 (11.9)	**2.27 (1.75–2.95)**	**<0.001**	1.43 (0.86–2.38)	0.170
**History of receiving FFP before 1974, n (%)**						
No	215 (69.8)	868 (84.3)	Ref		Ref	
Yes	93 (30.2)	162 (15.7)	**2.32 (1.73–3.11)**	**<0.001**	0.86 (0.46–1.62)	0.650
**History of receiving cryoprecipitate before 1974, n (%)**						
No	212 (69.1)	885 (85.8)	Ref		Ref	
Yes	95 (30.9)	146 (14.2)	**2.72 (2.02–3.66)**	**<0.001**	1.71 (0.91–3.22)	0.100
**History of receiving factor concentrates before 1993, n (%)**						
No	18 (5.8)	349 (33.7)	Ref		Ref	
Yes	290 (94.2)	688 (66.3)	**8.17 (4.99–13.38)**	**<0.001**	1.73 (0.94–3.18)	0.079

Abbreviations: n, number; OR, odds ratio; ref, reference; VWD, von Willebrand disease; FFP, fresh frozen plasma. * bivariable binary logistic regression. ** multivariable binary logistic regression. Bold cells represent significant differences in bivariable and multivariable logistic regression analysis.

**Table 3:** Bivariable and multivariable logistic regression analysis of the determinants of HCV-Ab prevalence in patients with hereditary bleeding disorders.

	Individuals with HCV-Ab, n (%)	Individuals without HCV-Ab, n (%)	OR (95% CI)	*p* Value *	Adjusted OR (95% CI)	Adjusted *p* Value **
**Sex, n (%)**						
Female	58 (6.6)	124 (20.9)	Ref		Ref	
Male	824 (93.4)	469 (79.1)	**3.76 (2.70–5.24)**	**<0.001**	2.04 (0.66–6.28)	0.214
**Birthyear, n (%)**						
Before 1997	881 (99.9)	400 (67.5)	Ref		Ref	
During and after 1997	1 (0.1)	193 (32.5)	**<0.01 (<0.01–0.02)**	**<0.001**	**0.01 (<0.01–0.12)**	**<0.001**
**Type of hereditary bleeding disorder, n (%)**						
Other disorders/VWD	122 (13.8)	259 (43.7)	Ref		Ref	
Hemophilia A	544 (61.7)	224 (37.8)	**5.16 (3.95–6.72)**	**<0.001**	1.83 (0.74–4.53)	0.188
Hemophilia B	216 (24.5)	110 (18.5)	**4.17 (3.04–5.71)**	**<0.001**	**2.91 (1.18–7.17)**	**0.020**
**Bleeding severity, n (%)**						
Mild	112 (14.5)	152 (39.3)	Ref		Ref	
Moderate	101 (13)	57 (14.7)	**2.41 (** **1.60–3.61)**	**<0.001**	**2.20 (1.33–3.65)**	**0.002**
Severe	561 (72.5)	178 (46.0)	**4.28 (3.18–5.75)**	**<0.001**	**5.99 (4.01–8.96)**	**<0.001**
**History of receiving packed cells before 1996, n (%)**						
No	388 (44.1)	438 (74.9)	Ref		Ref	
Yes	492 (55.9)	147 (25.1)	**3.78 (3.00–4.75)**	**<0.001**	**1.50 (1.01–2.23)**	**0.043**
**History of receiving FFP before 1996, n (%)**						
No	206 (23.4)	402 (68.7)	Ref		Ref	
Yes	674 (76.6)	183 (31.3)	**7.19 (5.69–9.09)**	**<0.001**	**2.21 (1.49–3.26)**	**<0.001**
**History of receiving cryoprecipitate before 1996, n (%)**						
No	185 (21.0)	410 (70.2)	Ref		Ref	
Yes	695 (79.0)	174 (29.8)	**8.85 (6.96–11.26)**	**<0.001**	**3.96 (2.56–6.12)**	**<0.001**
**History of receiving factor concentrates before 1997, n (%)**						
No	26 (2.9)	227 (46.9)	Ref		Ref	
Yes	856 (97.1)	313 (53.1)	**29.14 (19.10–44.45)**	**<0.001**	**3.01 (1.33–6.81)**	**0.008**

Abbreviations: n, number; OR, odds ratio; ref, reference; VWD, von Willebrand disease; FFP, fresh frozen plasma. * bivariable binary logistic regression. ** multivariable binary logistic regression.

**Table 4:** Analysis of determinants of HIV-Ab (confirmed) prevalence in patients with hereditary bleeding disorders.

	Individuals with HIV-Ab, n (%)	Individuals without HIV-Ab, n (%)	OR (95% CI)	*p* Value *
**Sex, n (%)**				
Female	0 (0)	182 (12.5)	Ref	
Male	18 (100)	1275 (87.5)	2.57 (0.34–19.36)	0.153
**Birthyear, n (%)**				
Before 1989	18 (100)	997 (68.4)	Ref	
During and after 1989	0 (0)	460 (31.6)	**0.12 (0.02–0.91)**	**0.002**
**Type of hereditary bleeding disorder, n (%)**				
Other disorders/VWD	0 (0)	381 (26.1)	Ref	
Hemophilia A	8 (44.4)	760 (52.2)	4.01 (0.50–32.18)	0.286
Hemophilia B	10 (55.6)	316 (21.7)	**12.06 (1.54–94.70)**	**0.004**
**Bleeding severity, n (%)**				
Mild	0 (0)	264 (23.1)	Ref	
Moderate	1 (5.6)	157 (13.7)	1.68 (0.10–27.07)	>0.999
Severe	17 (94.4)	722 (63.2)	6.22 (0.82–46.94)	0.055
**History of receiving packed cells before 1989, n (%)**				
No	18 (100)	1312 (90.7)	Ref	
Yes	0 (0)	135 (9.3)	0.54 (0.07–4.08)	>0.999
**History of receiving FFP before 1989, n (%)**				
No	18 (100)	1301 (89.9)	Ref	
Yes	0 (0)	146 (10.1)	0.50 (0.7–3.74)	0.713
**History of receiving cryoprecipitate before 1989, n (%)**				
No	18 (100)	1291 (89.3)	Ref	
Yes	0 (0)	155 (10.7)	0.46 (0.6–3.49)	0.712
**History of receiving factor concentrates before 1997, n (%)**				
No	0 (0)	303 (20.8)	Ref	
Yes	18 (100)	1151 (79.2)	4.74 (0.63–35.64)	0.149

Abbreviations: n, number; OR, odds ratio; ref, reference; VWD, von Willebrand disease; FFP, fresh frozen plasma. * Fisher’s exact test.

## Data Availability

The anonymized dataset of this study is available in Supplementary File S1.

## Supplementary Materials

The following supporting information can be downloaded at: https://www.mdpi.com/article/10.3390/pathogens12040555/s1, File S1: The anonymized study dataset.
